# The Effect of Supervisor Identification on Unethical Pro-Supervisor Behavior: The Moderating Role of Employability Perceptions

**DOI:** 10.3390/ijerph17249344

**Published:** 2020-12-14

**Authors:** Kangmin Lee

**Affiliations:** Institute for Future Talents, Hanyang University, 222, Wangsimni-ro, Seongdong-gu, Seoul 04763, Korea; kmrie@naver.com

**Keywords:** supervisor identification, employability perceptions, unethical pro-supervisor behavior (UPSB)

## Abstract

Under some employment circumstances, individuals in some organizations are willing to engage in unethical behaviors that benefit one’s own supervisors who have a great power to decide the levels of evaluation and compensation for each individual. In this study, two hypotheses were examined. First, based on social identification theory, we hypothesized that individuals’ feeling a sense of oneness with one’s own supervisors promote unethical pro-supervisor behaviors (UPSB). Second, based on a person–situation interactionist model, we hypothesized that this positive relationship is strengthened if the individual perceives lower levels of one’s own employability. Data were collected from 185 individuals of various types of organizations in South Korea. A time-lagged field study supported our hypotheses. In particular, [supervisor identification was positively related to UPSB. Furthermore, for individuals with a weaker employability perception, supervisor identification was positively related to UPSB.

## 1. Introduction

Umphress and Bingham [1] defined the concept of “unethical pro-organizational behavior (UPB)” to accurately understand and explain the problem in which individuals of an organization engage in unethical behaviors that undermine the desirable values of the larger society and country to which the organization belongs for the benefit of the organization to which they belong. And they proposed an integrated theoretical model that explains the causes, processes, and situational factors of unethical pro-organizational behavior based on various theories of social psychology [1]. In other words, they found the causes of unethical pro-organization behavior with the social exchange theory and the social identity theory, explained the process leading to unethical pro-organization behavior based on the neutralization theory, and recognized the surrounding circumstances and personal attributes that influence unethical pro-organization behavior based on the person–situation model of decision-making theory [2]. In addition, with an empirical study, it was revealed that the interaction between organizational identification and reciprocity belief is the cause of unethical pro-organizational behavior [3]. Since then, numerous researchers from both East and West have developed hypotheses based on theories of various social psychology and conducted field surveys of people in various regions, industries, and professions to find numerous variables that influence unethical pro-organizational behavior. For example, Miao et al. [4] identified that ethical leadership influences unethical pro-organizational behavior based on social learning theory, Chen et al. [5] identified that organizational identification is the cause of unethical pro-organizational behavior based on social identity theory, and Wang et al. [6] showed that high-quality social exchange relationships between the employees and the employers influence unethical pro-organizational behaviors based on the theory of social exchange.

We can easily find that individuals of the organization engage in unethical or illegal behaviors for their own supervisors. The most representative recent case is the Samsung’s illegal succession case. The executives and staffs of the Future Strategy Office, who had set mid-to-long-term strategies for the Samsung and manage the performances of each business, went beyond unethical behaviors to illegal behaviors for the benefit of Samsung Electronics Vice Chairman Lee Jaeyong [7]. To help vice chairman Lee Jaeyong’s illegal succession, they have systematically participated in illegal activities such as embezzling company money, bribing public officials, manipulating stock prices, and destroying evidence, and were convicted by a Korean court in return [8,9]. This case clearly shows that employees are willing to engage in unethical or illegal behaviors in order to benefit their supervisors. And this is the research topic we are interested in.

Recently, Johnson and Umphress [10] and Mesdaghinia et al. [11] have expanded the concept of unethical pro-organization behavior by specifying the beneficiaries of behaviors from the collective to supervisory individuals. In other words, they argued that individuals of an organization could engage in unethical behaviors (unethical pro-supervisor behavior) that undermine the desirable values of the larger society and country to which the organization belongs, for the benefit of their immediate supervisors. And according to their studies, it was found that the followers’ supervisor identification and supervisors’ bottom-line mentality are the causes of unethical pro-supervisor behaviors, and moral identity plays a role as a moderating variable that weakens the occurrence of unethical pro-supervisor behaviors. However, according to Umphress and Bingham’s [1] integrated theoretical model for unethical pro-organization behavior which reflects the person–situation interaction perspective and empirical studies on unethical pro-organization behavior, personal traits such as moral identity [10] and moral development [10] as well as situational conditions such as culture [1], bottom-line mentality climate perceptions [12] and industry competition perceptions [5] can control the occurrence of unethical pro-organizational behaviors. Similar to the studies on unethical pro-organizational behavior, it can be expected that situational conditions can control the occurrence of unethical pro-supervisor behavior. However, in recent empirical studies, the effects of situational conditions on unethical pro-supervisor behavior have not been discovered. Therefore, in this study, we would like to examine whether “Employability perceptions” moderate the occurrence of unethical pro-supervisor behaviors. Given the peculiarities of the Korean labor market with low employment flexibility [13], the employment is a resource that Korean workers do not want to lose [14,15,16]. In addition, individuals are more sensitive to the loss of resources they value and may experience stress when faced with the loss of valuable resources [17], and may take aggressive actions to conserve valuable resources [14,16]. Therefore, our research team predicted that employability perceptions would play a role in moderating the occurrence of unethical pro-supervisor behaviors. So, by conducting a field study for Korean workers in various industries and occupations, we want to answer two research questions.

**Research questions 1:** Will supervisor identification have a positive effect on the willingness to engage in UPSB?
**Research questions 2:** Will employability perceptions have a negative moderating effect the relationship between supervisor identification and the willingness to engage in UPSB?

## 2. Theoretical Development

### 2.1. Unethical Pro-Supervisor Behavior

Umphress et al. [3] established the concept of unethical pro-organization behavior in order to explain the social phenomenon of members (individuals) of an organization undermining or violating the values, laws, and norms of the society in which the organization exists for the benefit of the organization (and its members). And Umphress and Bingham [1] proposed an integrated theoretical model for unethical pro-organization behavior based on various theories such as social exchange theory [18,19], social identity theory [20], and a person–situation model of decision making [2]. Since then, numerous researchers have developed hypotheses based on various theories based on social psychology (e.g., social identity theory, social exchange theory, social learning theory), and found various causal variables and situational variables that affect unethical pro-organization behaviors through empirical studies [21]. For example, Chen et al. [5] found that organizational identification is the causal variable of unethical pro-organizational behavior based on the theory of social identity. And Wang et al. [6] showed that high social exchange relationships between employees and their employers motivate employees to perform unethical pro-organizational behaviors based on social exchange theory. In addition, Effelsberg et al. [22] found that transformational leadership influences unethical pro-organizational behavior based on social learning theory.

In recent years, some scholars have expanded the concept of unethical pro-organization behavior to explain the social controversy in which individuals commit unethical behaviors for their supervisors, who are different from the organization (or members of the organization) [10,11]. And Mesdaghinia et al. [11] defined unethical pro-supervisor behavior as “employees’ actions that further the interests of their leaders but violate ethical norms, values, or standards of proper employee conduct”. The beneficiaries of unethical pro-organizational behavior are organizations (and members of the organization), whereas the beneficiaries of unethical pro-supervisor behaviors are supervisors, entities that are distinct from members of the organization. That is, the beneficiaries of each action are different. Therefore, unethical pro-organizational behavior and unethical pro-supervisor behavior are distinct concepts [10]. Like unethical pro-organizational behavior, unethical pro-supervisor behavior must meet two conditions [5]. First, unethical pro-supervisor behavior is unethical in nature. Second, the intention of unethical pro-supervisor behavior is to benefit its supervisors. And, as can be seen in the measurement items, unethical pro-supervisor behavior includes two behaviors, act of commission (for example, employees could lie to help their supervisor or exaggerate their supervisor’s job performance to help him/her look good) and act of omission (for example, employees could withhold information that might damage their supervisor’s reputation to help their supervisor) [10]. And, with empirical research, it was found that organizational identification, supervisor identification, and leader’s bottom-line mentality are the causal variables of unethical pro-supervisor behaviors. It was also revealed that moral identity is a moderating variable that weakens the relationship between the causal variables and unethical pro-social behaviors [10,11]. Previous researchers emphasized the moderating effects of individual attributes.

### 2.2. Supervisor Identification

The self-identity means implicit, holistic, and consistent belief and feeling about “who am I”, and humans are basically animals that pursue identity [23,24]. Identification contributes to the formation of self-identity. In other words, the positive/negative cognitive relationship that an individual has toward the object they identify with provides the basis for self-identity [25,26]. According to the targets, levels of self, and levels of abstraction to identify in the organizational context, identification can be classified into person identification, relational identification, and organization identification [27]. Here, if the subject to be identified is limited to the supervisor, which is the most important relationship in the workplace [28], supervisor identification can be defined as “the perceived oneness with one’s supervisor” [10].

**Figure 1:**
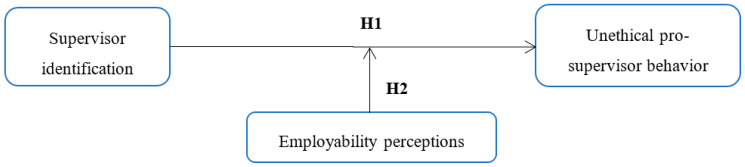
Hypothesized model.

The oneness with the supervisors perceived by the employees not only makes them value and positively evaluate the attributes of the supervisors but also makes them define themselves with the attributes of the supervisors [10,27]. And employees with high levels of supervisor identification add value to their relationship with their supervisors and develop a desire to maintain, expand, and improve that relationship [28]. So, employees can even take action that will help their supervisors far beyond what is officially required for the job [28]. In an empirical study of Chinese workers, supervisor identification was found to have a positive effect on organizational citizenship behavior [28], and, in a study on Korean workers, supervisor identification was found to have a positive effect on intrinsic motivation [29]. Furthermore, employees with high levels of supervisor identification can internalize their supervisors’ goals and even engage in unethical behaviors to help those [10]. In empirical studies of US and Korean workers, studies consistently show that supervisor identification can be a direct cause of unethical pro-supervisor behavior. Based on the arguments presented above, we propose the following (see Figure 1):

**Hypothesis** **1:** *Supervisor identification will be positively related with the willingness to engage in UPSB.*

### 2.3. Employability Perceptions

Putting previous studies dealing with various aspects of employability together, employability can be defined as “the possibility of accessing a suitable job or to remain employed in a social, economic, cultural, and technological context” [30]. A number of existing empirical studies have only dealt with the individual aspects of employability, and overlooked aspects of organizational strategy, government and education policies [30]. And when measuring the employability, we only relied on the self-evaluation without considering the temporal aspects [30]. In line with these prior studies, employability perceptions can be specifically defined as “the individual’s perception of his or her possibilities of obtaining and maintaining employment” [31]. Rothwell and Arnold [32] developed a tool to measure employability perceptions by conducting a survey of 200 HR professionals in UK and analyzing the data. According to their research, the employability was found to have two sub-dimensions: internal employability and external employability.

Recognizing one’s employability as a low means that one’s job-related skills, experiences, networks, personal characteristics, and understandings of the labor market are lacking [33]. In other words, they perceive their labor competitiveness as a low in the labor market. In general, individuals’ perceptions of the situations affect the individuals’ emotions, attitudes, and behaviors [34,35,36,37]. Since the employment is an important issue that gives meaning to individual employees, it affects the individuals’ physical and mental health [34,38]. In particular, the perception that one’s own employability is low leads to the fear of losing a job, which adversely affects the individual’s physical and mental health [34,39,40]. These fears motivate individuals to overcome their fears [41,42]. For example, employees who perceive that they are at risk of being expelled from the group may try to prove their worth and improve their status by contributing more to group performances [41,42]. In other words, individuals who feel fear of loss or exclusion can participate in pro-group behaviors for the benefits of their groups. And, according to some empirical studies, these individuals go further into unethical behaviors that contribute to the interests of the group and organization (and members of the organization) to which they belong, but undermine higher values [14,42]. Considering that the supervisors’ job is to create and manage the groups’ performances, and that the supervisors’ authorities are to evaluate and compensate for each individual’s contributions to the groups’ performances [43], it can be inferred that the individuals who feel fear of loss or exclusion can even take unethical behaviors for their supervisors who are a more specific object than the groups.

And the self-depletion theory makes it easier to understand the process by which these negative perceptions affect unethical behaviors [44]. The self-control refers to “the capacity to alter or override dominant response tendencies, and to regulate behavior, thoughts, and emotions” [45,46], which is a finite resource that can be depleted in a short period of time [46,47]. And the persistent stress leads to the depletion of emotional resources [48], which makes the self-control difficult [49]. Considering the definition of stress [50], an individual who perceives own employability as a low can be considered to be under stress. Therefore, individuals who perceive that they are unlikely to be employed continue to experience stress, exhaust their finite emotional resources, and lose the power to consider, judge, and suppress the ethics of behaviors. In other words, individuals who have sufficient emotional resources can fully take into account ethical standards when deciding what to do to deal with the crisis they are facing, whereas individuals who lack emotional resources are buried only in resolving the crisis and lose the space to examine the ethics of behaviors. Recent empirical studies [51,52] proved that the stress such as job insecurity leads to the self-depletion, resulting in the unethical pro-organizational behavior.

In addition, according to Umphress and Bingham’s [1] integrated theoretical model of unethical pro-organization behavior that reflects the person–situation interaction perspective, situations such as organizational culture perceived by individuals can play a role in controlling the impact of perceptions such as positive social exchange and organizational identification on the unethical pro-organizational behavior. In several empirical studies, it has been found that the perception of employability actually plays a role as a moderating variable in the effect of fear on behaviors. For example, in a study of white-collar jobs in Sweden, fear of job loss was found to be related to decreased speeches and loyalties, and the perception of employability played a role in strengthening the relationship between them [53]. In this study, employability perception was not considered as a factor that directly influences unethical pro-supervisor behavior as a result of the self-control failure resulting from the self-depletion or as a means to overcome the crisis of job loss. Instead, we understood that the employability perception as a context variable perceived by individuals, and predicted that a low level of employability perception would reinforce unethical pro-supervisor behavior of employees with a high level of supervisor identification. Based on the arguments presented above, we propose the following (see Figure 1):

**Hypothesis** **2:** *The positive relationship between supervisor identification and the willingness to engage in UPSB will be stronger when Employability Perceptions are lower rather than higher.*

## 3. Method

### 3.1. Sample and Procedure

Data for this study were collected via Google’s online field survey of full-time and part-time employees working in various types of organizations in South Korea. We took a number of steps to reduce common method biases [54]. First, we informed participants that the responses to our survey questions would be processed to be anonymous, so they could frankly express their opinions without the fear of upcoming consequences. Second, our survey had two parts which were approximately 4 weeks apart from one another. Cell phone numbers of participants were asked at both times. Some data from participants who showed a discrepancy were discarded. Participants who answered all questions in two surveys were paid USD 3.0 for their participation. Third, some questions to measure the social desirability were included in our survey because of the sensitive nature of our dependent variable [10].

At first survey, we sent text messages with URL to 500 individuals, inviting them to participate in a mobile survey (docs.google.com). A total of 272 provided responses to the first survey (response rate: 54.4%). Of these, 3 were dropped because they typed their cell phone numbers incorrectly. We used cell phone numbers of participants as a key value to match the responses at first survey and second survey. At second survey, approximately 4 weeks later, the participants who had completed the first survey were invited to ask all questions in the second survey. A total of 191 provided responses to the second survey (response rate: 71.0%). Of these, 6 were dropped due to typing errors of their own cell phone numbers. This led to a final sample size of 185, of whom 40.5% were female and 82.7% were in their 30 s and 40 s and 60.5% have worked for more than 5 years. Additionally, they worked in various types of jobs (e.g., production, sales, planning, accounting, HR, and research and development) in different industries (e.g., manufacturing, retail, IT, finance, public service, and education). The full-time employees of participants were 86.5%. The demographic characteristics of respondents are described in Table 1.

### 3.2. Measures

Participants rated their supervisor identification at the first survey and their own unethical pro-supervisor behavior and employability perception at the second survey. All items were measured on 5-point Likert scale ranging from 1 = strongly disagree to 5 = strongly agree unless stated otherwise. All of the items appear in Appendix A, Appendix B and Appendix C. At the first survey, we also asked participants about the social desirability and their demographic characteristics such as gender, age, tenure, jobs, industries, and employment status.

#### 3.2.1. Supervisor Identification

Supervisor identification was measured with five items developed and validated by Mael and Ashforth [55] to refer to the supervisor rather than the organization [10]. Participants rated their agreements with items such as “When someone criticizes my supervisor, it feels like a personal insult” (1 = strongly disagree to 5 = strongly agree). Cronbach’s α for this scale was 0.864.

#### 3.2.2. Employability Perceptions

Employability perceptions were measured with eleven items developed and validated by Rothwell and Arnold [32]. Self-perceived employability has two components: internal employability (relating to the internal labor market) and external employability (relating to the external labor market). Four items were designed to reflect internal employability and the others were designed to reflect external employability [32]. Participants rated their agreements with items such as “Even if there was downsizing in this organization, I am confident that I would be retained” and “The skills I have gained in my present job are transferable to other occupations outside this organization” (1 = strongly disagree to 5 = strongly agree). Cronbach’s α for the internal employability and the external employability were 0.604 and 0.841, respectively.

#### 3.2.3. Unethical Pro-Supervisor Behavior

Unethical pro-supervisor behavior was measured with six items developed and validated by Johnson and Umphress [10]. The past studies on unethical pro-supervisor behavior measured unethical pro-supervisor behavior using unethical pro-organizational behavior scale [1] to refer to the supervisor (or leader) rather than the organization [10,11]. Participants rated their agreements with items such as “Because it was needed, I have concealed information from others that could be damaging to my supervisor” (1 = strongly disagree to 5 = strongly agree). Cronbach’s α for this scale was 0.824.

#### 3.2.4. Control Variables

We controlled for the social desirability because previous studies suggest the impression management bias may occur when individuals answer sensitive questions such as UPB and unethical pro-supervisor behavior (UPSB) [1,10]. We used eight items developed and validated by Stober [56]. Moreover, we controlled participants’ gender, age, tenure, jobs, industries, and employment status because previous research has shown that demographic characteristics may influence unethical behaviors [11].

### 3.3. Data Analysis

First, we conducted confirmatory factor analysis using IBM AMOS 18.0 to verify the validity of the measures in our research model. Next, we conducted reliability analysis with Cronbach’s α using IBM SPSS 18.0 to examine the reliability of the measures in our research model. Then, we conducted a correlation analysis to determine the association between the variables in our model. Finally, we conducted causal analysis and moderating effect analysis using SPSS Macro Model 1 [57] to test our hypotheses.

## 4. Results

Before conducting confirmatory factor analysis and hypotheses tests, we checked for possible common method variance with Harman’s single-factor test [58]. According to this approach, common method variance is present if a single factor accounts for the majority of the covariance in the variables. We found the first factor explaining 33.177% of the total variance from the factor analysis, implying that common method variance was not present.

### 4.1. Confirmatory Factor Analysis

We conducted CFA using IBM AMOS 18.0 to demonstrate the distinction between supervisor identification, internal employability perception, external employability perception and unethical pro-supervisor behavior. We compared the four-factor model to a series of nested models as supported with significant increases in Chi square at a time [59]. The four-factor model for supervisor identification, internal employability perception, external employability perception, and unethical pro-supervisor behavior demonstrated a better fit to the data (χ^2^ (110) = 160.041; RMR = 0.058; GFI = 0.904; NFI = 0.886; CFI = 0.961; RMSEA = 0.050, Δχ^2^ = 18.986; *p* < 0.05) compared to a three-factor model in which internal and external employability perception items load onto one factor and supervisor identification items load onto a second factor and the unethical pro-supervisor behavior items load onto a third factor. The three-factor model for supervisor identification, employability perceptions, and unethical pro-supervisor behavior demonstrated a better fit to the data (χ^2^ (113) = 217.000; RMR = 0.076; GFI = 0.861; NFI = 0.845; CFI = 0.918; RMSEA = 0.071, Δχ^2^ = 165.113; *p* < 0.05) compared to a two-factor model in which supervisor identification and employability perception items load onto one factor and the unethical pro-supervisor behavior items load onto a second factor. The two-factor model demonstrated a worse fit (χ^2^ (115) = 547.225; RMR = 0.122; GFI = 0.705; NFI = 0.610; CFI = 0.659; RMSEA = 0.143, Δχ^2^ = 159.673; *p* < 0.05). We then compared the two-factor model to a single-factor model in which all items load onto one factor. The single-factor model demonstrated the worst fit (χ^2^ (116) = 706.898; RMR = 0.148; GFI = 0.654; NFI = 0.496; CFI = 0.534; RMSEA = 0.166).

Therefore, we concluded the measurement model to support the distinction between supervisor identification, internal employability perception, external employability perception, and unethical pro-supervisor behavior was acceptable.

### 4.2. Hypotheses Tests

Table 2 shows means, standard deviations, reliabilities, and inter-correlations among the variables in this study. We tested Hypothesis 1 and 2 using the hierarchical regression (see Table 3).

We tested Hypothesis 1, which stated that supervisor identification predicts unethical pro-supervisor behavior. To test the causal effect of supervisor identification on unethical pro-supervisor behavior, we took two steps in our research model. In Step 1, we included the control variables. In Step 2, we included supervisor identification as predictor of the unethical pro-supervisor behavior. As the results for Step 2 in Table 3 show, the main effect of supervisor identification on unethical pro-supervisor behavior was significant (B = 0.197, SE = 0.066, *p* < 0.01), therefore supporting Hypothesis 1.

**Figure 2:**
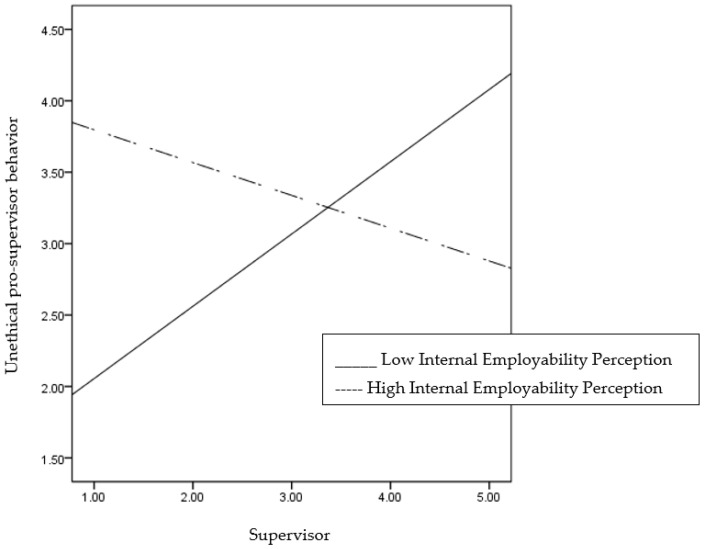
Moderating effect of internal employability perception on the relationship between supervisor identification and UPSB.

Next, we tested Hypothesis 2 which stated that the positive relationship between supervisor identification and unethical pro-supervisor behavior would be stronger when the employability perceptions are low rather than high. To test the moderating effect of employability perceptions between supervisor identification and unethical pro-supervisor behavior, we used Macro 3.5 for SPSS developed by Hayes [57]. Specifically, model number 1 was selected, the confidence interval was set to 95%, and the number of bootstrap samples was set to 5000. Then, unethical pro-supervisor behavior was entered in the Y variable, supervisor identification was entered in the X variable, and employability perceptions were in Moderator variable W. As the results in Table 4 show, the interaction between supervisor identification and internal employability perception was significantly related to unethical pro-supervisor behavior as predicted (B = −0.2471, SE = 0.0856, *p* < 0.01). To further analyze the nature of the interaction, we plotted the relationship between supervisor identification and unethical pro-supervisor behavior at ±1 SD of the internal employability perception (Figure 2). We also statistically explored the nature of the interaction by conducting the simple slopes analysis [60,61]. The results indicate that supervisor identification is significantly positively related to unethical pro-supervisor behavior for employees with a low level of internal employability perception (B = 0.506, SE = 0.168; *p* < 0.01); however, the relationship was not significant for employees with a high level of internal employability perception (B = −0.230, SE = 0.151; ns). On the other hand, the interaction between supervisor identification and external employability perception was not significantly related to the unethical pro-supervisor behavior against our prediction (see Table 5). Thus, Hypothesis 2 is partially supported.

## 5. Discussion

By demonstrating that individuals of the organization participate in unethical behaviors for their superiors even in Korea which belongs to the East, it confirmed that unethical pro-supervisor behaviors are universal behaviors that are free from cultural differences in the countries and societies in which the study objects (subjects) belong. In addition, it was demonstrated that individuals’ supervisor identifications and employability perceptions had an effect on the occurrence of unethical pro-supervisor behaviors. It was confirmed that the two hypotheses developed with the field study were supported. First, it was found that the supervisor identification had a statistically significant positive (+) effect on unethical pro-supervisor behavior. In other words, Employees who strongly identify themselves with their supervisors are willing to perform unethical behaviors taboo in their society for their supervisors. This is consistent with the results of previous studies [10] that proved unethical pro-supervisor behaviors for research subjects belonging to the West. Second, it has been newly revealed that the employability perceived by individuals play a negative (-) moderating role in the relationship between the supervisor identification and the unethical pro-supervisor behavior. Specifically, it was confirmed that the internal employability perceived by individuals plays a negative (-) moderating role in the relationship between the supervisor identification and the unethical pro-supervisor behavior. On the other hand, it was found that the external employability perception did not play a moderating role in the relationship between the supervisor identification and the unethical pro-supervisor behavior. In other words, individuals who perceive in their surrounding situation that the organization’s internal employability is low are more likely to engage in unethical pro-supervisor behaviors, while individuals who recognize that the organization’s internal employability is high are less likely to engage in unethical pro-supervisor behaviors.

## 6. Conclusions

### 6.1. Theoretical Implications

This study gives three academic contributions. First, it confirmed that it is a common behavior that individuals of the organization participate in unethical behaviors for their supervisors. Previous studies surveyed people working or studying in the United States belonging to the West [10,11]. On the other hand, this study surveyed full-time and part-time employees who work in various organizations in Korea belonging to the East. In addition, by demonstrating that unethical pro-supervisor behaviors can be observed in the East, it was shown that the variable of organizational behavior called unethical pro-supervisor behavior can be a variable with universal validity. With this, it provided the basis for active studies on unethical pro-supervisor behaviors in the East.

Second, it contributed academically to the empirical studies on the supervisor identification by demonstrating the negative effects that can be caused by the supervisor identification. Up to now, the supervisor identification has been known to have a beneficial effect on variables of various levels (individual, group, organization) that are positively evaluated in the field of organizational behavior [10]. For example, the supervisor identification has been confirmed to have a positive effect on the self-concept, the intrinsic motivation [29], and the organizational citizenship behavior [28]. However, with this study, it was revealed once again that supervisor identification can be a direct cause of unethical pro-supervisor behavior [11] that affects the intention to turn over. Thus, this provided the justification for paying attention not only to the positive effect but also the negative effect in the study dealing with the supervisor identification.

Third, it contributed to the studies of unethical pro-supervisor behaviors by identifying the new variable that plays a moderating role in the relationship between supervisor identification and unethical pro-supervisor behavior. This study, approaching from the perspective of individual–situation interaction, has shown that employees who strongly identify with their supervisors can engage in unethical behaviors taboo in their societies for their supervisors, and that these negative consequences can be reinforced when their subordinates perceive low levels of internal employability. These findings suggest that unethical pro-supervisor behavior can be reduced if the perceptions that the organization’s internal employability is not low are created and spread among the individuals of the organization. We can find academic contributions in that we demonstrated the effect and importance of the situational awareness in the studies of unethical pro-supervisor behaviors.

### 6.2. Practical Implications

This study gives some practical implications. First, organizations (especially companies) must conduct thorough precautionary activities to prevent the occurrence of employees’ unethical pro-supervisor behavior by accurately diagnosing the levels of employees’ supervisor identification and maintaining appropriate levels. This is because when unethical behaviors within the organization are disclosed outside the organization, companies face many difficulties [21]. Specifically, customers turn their face away from the products and services of unethical companies and do not consume those [62]. And employees who work in unethical companies experience decreased job satisfaction [63], hindered organizational commitment [64] and even become willing to leave the organizations [11]. Moreover, excellent talents do not apply to such unethical companies [65]. In addition, some investors may hesitate to invest in unethical companies [66]. Therefore, companies should diagnose the levels of employees’ supervisor identification on a regular or irregular basis and respond based on this. Through notice, guidance, and education, the risk of adverse effects that can be caused by high levels of employees’ supervisor identification should be communicated to and shared with employees in the organizations.

Second, organizations (especially companies) should also pay attention to the levels of employability perceived by their employees. In this study, it was found that employees’ low levels of employability perceptions can strengthen the relationship between the supervisor identification and the unethical pro-supervisor behavior. In particular, the finding that this phenomenon occurs in employees who have low levels of the internal employability perceptions has great implications. Employees who perceive their internal employability as low think that they are not competitive in the internal job markets [33] and can be obsessed with the fear that they may lose their internal jobs in the form of job transfers, department transfers, etc. [34]. And in order to solve this difficult situation, it has been confirmed that employees can even take unethical actions for the benefits of supervisors who can evaluate themselves and perform personnel actions on them. Therefore, companies should diagnose the levels of employees’ employability perceptions and take appropriate actions so that individuals of the organization do not lose confidence and become fearful and engage in unethical behaviors. For example, we can consider implementing training programs to improve the job competency for employees who have low levels of internal employability perceptions.

### 6.3. Limitations and Future Directions

This study has several limitations. First, since all variables included in the study model were measured by the self-reporting method, they cannot be free from the issue of common method bias [54]. However, while conducting this study, several possible steps have been taken to reduce the common method bias. First, the timing of the measurement of the dependent variable and the measurement of the independent variables were different. At the risk of reducing the response rate, the dependent variable and the independent variables were measured separately at 4-week intervals. Second, the social desirability was included in the list of control variables in consideration of the nature of the unethical pro-supervisor, and the influence of the social desirability on the unethical pro-supervisor behavior was controlled in the process of analyzing data. Third, a notice that guarantees anonymity was introduced in the beginning of the surveys so that respondents could respond honestly, and respondents were allowed to participate in the surveys online. Fourth, the subject of this study and the variables included in this research model were expressed abstractly so that it is difficult to guess them. In future research, it is possible to consider a method of measuring employees’ supervisor identification through the observations instead of the questionnaires, and a method of obtaining data on employees’ unethical pro-supervisor behaviors that actually occurred in the organizations after obtaining consents from the HR managers of the organization. With these efforts, I hope to be free from the issue of common method bias caused by the self-reporting method.

Second, the hypothesis partially supported that the employment perceptions would moderate the relationship between the supervisor identification and the unethical pro-supervisor behavior. To be specific, the moderating effect of the internal employability perception constituting the employability perceptions was verified, while the moderating effect of external employability perception was not verified. The reason for this result can be found in the source of the data used in the analysis. This study took employees working in Korea as the target of the research. However, Korea’s employment environment has different characteristics. Korea’s employment flexibility is very low among OECD (The Organisation for Economic Co-operation and Development) countries with similar economies [13]. In other words, employees working in Korea have a very low tendency to leave their own organization to change jobs. Therefore, it is possible that Korean workers have little interest in the external employability and, furthermore, are unconsciously not even aware of the external employability. In future studies, it is expected that interesting research results can be obtained by conducting research on employees working in countries with a similar economic scale to Korea but with high employment flexibility and comparing the results with this study results.

Third, a sufficient number of sample data were not collected, so further analysis to bring about comprehensive findings could not be conducted. For example, in this paper, sample data were collected from employees working in various industries, but we could not test whether there were differences in hypotheses by industry characteristics. We only use the demographic characteristics of respondents as control variables when testing hypotheses. In future studies, it is expected that a sufficient number of sample data will be collected to verify whether there are differences in the study results based on the demographic characteristics of the respondents to find comprehensive findings. In addition, it is necessary to conduct comparative studies between countries to see if the research results are different depending on the region, country, and culture. In other words, it is necessary to comprehensively deal with general results that can be found in common despite differences in regions, countries, and cultures, and special results that vary by differences in regions, countries, and cultures. In other words, it is expected to comprehensively deal with universal research results that appear in common despite differences in regions, countries, and cultures, as well as special research results arising from differences in regions, countries and cultures.

## Figures and Tables

**Table 1:** Demographic characteristics of respondents.

Characteristics	Categories	Frequencies	Ratios
Gender	Male	110	59.5
	Female	75	40.5
Age	20 s	21	11.4
	30 s	88	47.6
	40 s	65	35.1
	50 s	11	5.9
Employment period	Less than 3 years	41	22.2
	3–5 years	32	17.3
	5–10 years	54	29.2
	More than 10 years	58	31.3
Industry	Manufacturing	26	14.1
	Electricity, gas, steam and air conditioning supply	2	1.1
	Construction	5	2.7
	Wholesale and retail trade	15	8.1
	Transportation and storage	5	2.7
	Accommodation and food service activities	1	0.5
	Information and communication	36	19.5
	Financial and insurance activities	11	5.9
	Real estate activities	1	0.5
	Professional, scientific and technical activities	32	17.3
	Public administration and defense; compulsory social security	10	5.4
	Education	21	11.4
	Human health and social work activities	11	5.9
	Arts, sports and recreation related services	6	3.2
	Membership organizations, repair and other personal services	2	1.1
	Activities of extraterritorial organizations and bodies	1	0.5
Employment status	Full-time	160	86.5
	Part-time	25	13.5

**Table 2:** Means, standard deviations, reliabilities, and inter-correlations.

Measures	M	SD	α	1	2	3	4	5
1. Social desirability	2.815	0.765	0.694	1				
2. Supervisor identification	2.684	0.842	0.864	−0.048	1			
3. Internal employability perception	3.611	0.595	0.604	0.046	0.287 ***	1		
4. External employability perception	3.270	0.768	0.841	−0.062	0.095	0.321 ***	1	
5. Unethical pro-supervisor behavior	2.970	0.736	0.824	0.173 *	0.279 ***	0.196 **	0.106	1

N = 185; * *p* < 0.05; ** *p* < 0.01; *** *p* < 0.001.

**Table 3:** Hierarchical regression results.

Variables	Unethical Pro-Supervisor Behavior	
	Step 1	Step 2
Control variable		
Social desirability	0.182 *	0.187 **
Predictor variables		
Supervisor identification		0.197 **
Internal employability perception		0.060
External employability perception		0.176 *
F	2.388 *	3.912 ***
R^2^	0.079	0.176
Adjusted R^2^	0.046	0.131
ΔR^2^	0.079 *	0.097 ***

N = 185; * *p* < 0.05; ** *p* < 0.01; *** *p* < 0.001.

**Table 4:** Results of the moderating effect of internal employability perception in the relationship between supervisor identification and unethical pro-supervisor behaviors (UPSB).

Variables	B	SE	t	95%	
				LLCI	ULCI
Supervisor identification	1.1264	0.3228	3.4896 ***	0.4895	1.7634
Internal employability perception	0.8090	0.2430	3.3294 **	0.3295	1.2884
Supervisor identification × Internal employability perception	−0.2471	0.0856	−2.8874 **	−0.4160	−0.0782

N = 185; * *p* < 0.05; ** *p* < 0.01; *** *p* < 0.001.

**Table 5:** Results of the moderating effect of external employability perception in the relationship between supervisor identification and UPSB.

Variables	B	SE	t	95%	
				LLCI	ULCI
Supervisor identification	0.1820	0.2800	0.6499	−0.3705	0.7345
Internal employability perception	0.0315	0.2349	0.1340	−0.4319	0.4949
Supervisor identification × Internal employability perception	0.0172	0.0846	0.2035	−0.1497	0.1841

## Appendix A. Supervisor Identification Scale Items

1. When someone criticizes my supervisor, it feels like a personal insult.
2. I am very interested in what others think about my supervisor.
3. When I talk about my supervisor, I usually say “my supervisor”.
4. My supervisor’s success is my success.
5. When someone praises my supervisor, it feels like a personal compliment.

## Appendix B. Employability Perceptions Scale Items

1. Even if there was downsizing in this organization, I am confident that I would be retained.
2. My personal networks in this organization help me in my career.
3. I am aware of the opportunities arising in this organization even if they are different to what I do now.
4. Among the people who do the same job as me, I am well respected in this organization.
5. The skills I have gained in my present job are transferable to other occupations outside this organization.
6. I could easily retrain to make myself more employable elsewhere.
7. I have a good knowledge of opportunities for me outside of this organization even if they are quite different to what I do now.
8. If I needed to, I could easily get another job like mine in a similar organization.
9. I could easily get a similar job to mine in almost any organization.
10. Anyone with my level of skills and knowledge, and similar job and organizational experience, will be highly sought after by employers.
11. I could get any job, anywhere, so long as my skills and experience were reasonably relevant.

## Appendix C. Unethical Pro-Supervisor Behavior Scale Items

1. Because it was needed, I have concealed information from others that could be damaging to my supervisor.
2. Because my supervisor needed me to, I have not revealed to others a mistake he/she made that would damage his/her reputation.
3. Because it helped my supervisor, I have exaggerated the truth about my supervisor’s performance to others.
4. Because it benefited my supervisor, I have withheld negative information about my supervisor’s performance from others.
5. Because it helped my supervisor, I have misrepresented the truth to make my supervisor look good.
6. Because my supervisor needed me to, I spoke poorly of another individual who was a problem for my supervisor.
